# Extents of Predictors for Land Surface Temperature Using Multiple Regression Model

**DOI:** 10.1155/2020/3958589

**Published:** 2020-08-06

**Authors:** R. M. Yuvaraj

**Affiliations:** UGC-SRF, Department of Geography, Queen Mary's College, Chennai, India

## Abstract

Land surface temperature (LST) is a key factor in numerous areas such as climate change, land use/land cover in the urban areas, and heat balance and is also a significant participant in the creation of climate models. Landsat data has given numerous possibilities to understand the land processes by means of remote sensing. The present study has been performed to identify the LST of the study region using Landsat 8 OLI/TIRS satellite images for two time periods in order to compare the data. The study also attempted to identify and predict the role and importance of NDVI, NDBI, and the slope of the region on LST. The study concludes that the maximum and minimum temperatures of 40.44 C and 20.78 C were recorded during the November month whereas the maximum and minimum LST for month March has increased to 42.44 C and 24.57 C respectively. The result indicates that LST is inversely proportional to NDVI (−6.369) and slope (−0.077) whereas LST is directly proportional to NDBI (+14.74). Multiple linear regression model has been applied to calculate the extents of NDVI, NDBI, and slope on the LST. It concludes that the increase in vegetation and slope would result in slight decrease in temperature whereas the increase in built-up will result in a huge increase in temperature.

## 1. Introduction

Land surface temperature (LST) is an indispensable factor in the physics of land surface processes: it plays the most significant role in the transfer of energy and water from the ground to the atmosphere [1]. LST is regulated by radiation from the sun and the exchange of heat between land and atmosphere [2]. Therefore, the spatial and temporal distributions of LST reveal the changes in climatic factors and the characteristics of the land surface. A detailed study of the spatial and temporal changes of LST is essential to different research fields which include surface energy budgeting [3, 4], urban climate, vegetation [5, 6], and hydrology [7, 8]. Currently, remote sensing satellite data is the most suitable way to study the spatial and temporal variations of LST [9]. Elevation was considered as the most impactful variable effect on LST [10–12]. Extensive urbanization, which modifies the land use and land cover (LULC) [13], alters the energy balance and makes built-up land hotter than its surrounding areas where the built-up land is less. For the purpose of extraction of LST can be done with the help of remote sensing which has ample range of sensors, like Landsat 4 and 5 (TM), 7 (ETM+), 8 (TIRS 1 and 2), Moderate Resolution Imaging Spectroradiometer (MODIS), Advanced Spaceborne Thermal Emission and Reflection (ASTER), and Advanced Very High Resolution Radiometer (AVHRR) [14], and the study also confirms that the remote sensing provides accurate temperature value than ground station [15]. Land use and land cover (LU/LC) of a given area could be utilized for estimating the amount of LST because the temperature varies based on the different land use. The natural process and anthropogenic activities are responsible causes of changes in the LU/LC of an area which also controls the LST of that area. LST has a direct relation with the local climate. If the value of LST changes, the local climate of the area also changes. It is an essential phenomenon to be investigated to study the local climate which would be used for proper future planning. Hence, many researchers had calculated LST using a variety of algorithms and techniques. Vegetation can effectively influence LST by selectively absorbing and reflecting solar radiation energy and regulating latent and sensible heat exchange. Normalized difference vegetation index (NDVI) is a vegetation identifier in the area that is purposefully utilized in the study to find the relationship with LST [16–20]. It is familiar that the air temperature reduces with an increase in altitude in the troposphere of the Earth's atmosphere, and this reduction is termed as lapse rate. The lapse rate is the decrease in temperature with an increase in height, at any given location, along the same column of air above the Earth surface, i.e., in the vertical direction. The lapse rate varies from 5°C to 10°C per 1000 m based on the moisture conditions. It means elevation is a direct response to LST [21]. There is a number of researches that have proved that built-up land can accelerate the surface temperature of the land [5, 22]. The study has used a normalized difference built-up index (NDBI) which is considered as a significant technique for the delineation of built-up land [23] in the given area. The current study has used Landsat 8 data for finding the LST, Normalized Differential Vegetation Index (NDVI), and Normalized Differential Built-Up Index (NDBI) [24]. The main objectives of the study are to find the LST for the months of March and November, to find the NDVI, NDBI, and slope. Finally, multiple linear regression model has been created for identifying the predictor and its extent for the LST. This model helps to understand how much the LST changes when the NDVI, NDBI, and slope change. Regression analysis of LST has been performed by Aakriti & Ram 2015 with NDVI, which is strongly determinant.

### 1.1. Study Area

**Figure 1:**
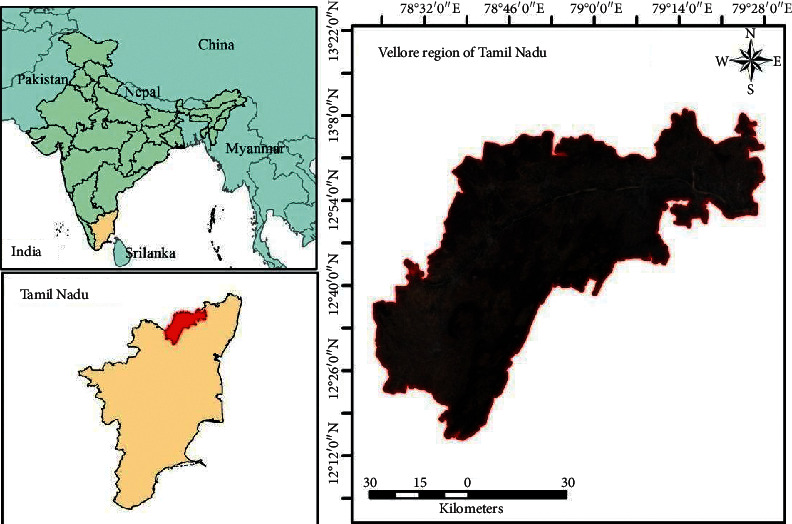
Study area.

The study area of Vellore region is located in the northern parts of Tamil Nadu, India, between 12°14′45″N to 13°8′52″N and 78°23′45″ to 79°27′45” (Figure 1). This region comes under the Vellore district administration of Tamil Nadu. The total area of the region is 4,708 Sq. Km with a total population of 2,464,475 as per the 2011 census. The average sunshine hours of the region are 2762 hours per year and the average rainfall of the region is 795 mm, out of which 67 percent is received during northeast monsoon season.

The average annual temperature is 27.3 C with maximum temperature being 38.2 C recorded during May and minimum temperature recorded during January. The study region is considered the hottest in Tamil Nadu because it is located in the Eastern Ghats surrounded by mountains. The detection of the extent of land surface temperature would be useful for adopting mitigation measures.

### 1.2. Data

For finding the land surface temperature (LST) of the study region, Landsat 8 Operational Land Imagers (OLI) and Thermal Infrared Sensor (TIRS) images with 143 paths and 51 rows have been downloaded from USGS (United States Geological survey) [25] which provides 11 bands with different wavelengths. Advanced Spaceborne Thermal Emission and Reflection Radiometer (ASTER), Global Digital Elevation Model (GDEM) has been downloaded from the same website [26]. Details of the acquired satellite data and its characteristics are given in Table 1.

### 1.3. Methodology

The satellite images have gone through preprocessing of extracting the images based on the study area with the help of extract by mask tool in ArcGIS 10.2 software. After preprocessing, the study area of the Vellore region and its corresponding satellite images are obtained for the data processing and analysis. In this study, only band 10, band 4, and band 5 are used for calculating the land surface temperature (LST). Band 10 is a Thermal Infrared Sensor (TIRS) with a wave length of 10.60 to 11.19; band 4 is red with wavelength of 0.64 to 0.67, and band 5 is Near Infrared (NIR) with wavelength of 0.85 to 0.88 [27]. The following algorithm has been utilized to retrieve the land surface temperature of the study region. The initial procedure of retrieving land surface temperature is calculating the TOA (Top of Atmospheric) spectral radiance:

(1) $$\begin{array}{c} \text{TOA}\left(L\right)=M_{L}*Q_{\text{cal}}+A_{L}-O_{i}, \end{array}$$

where *M*_*L*_ represents the multiplicative rescaling factor of specific band [25], *Q*_cal_ is quantized and calibrated standard product pixel values (DN), *A*_*L*_ is Additive rescaling factor of specific band [25], and *O*_*i*_ is the band correction [25, 28]. *M*_*L*_, *O*_*i*_, and *A*_*L*_ are the metadata of the Landsat image. The value is given in Table 2. The next step is the conversion of spectral radiance to brightness temperature (TB) using the constant values provided in the metadata. The following equation has been adopted to convert the reflectance to brightness temperature:

(2) $$\begin{array}{c} \text{TB}=\;\frac{K_{2}}{\text{In }\left[\left(K_{1}/L\right)+1\right]}-273.15, \end{array}$$

where *K*_1_ and *K*_2_ are the thermal conversion constants for specific bands [25] given in Table 2, *L* is the top of the atmosphere. In order to obtain the results in Celsius, the radiant temperature is adjusted by adding the absolute zero (−273.15 C).

Land surface emissivity (LSE) estimation from the NDVI method, LSE factor must be known in order to calculate the LST, since the LSE is a proportionality factor that scales blackbody radiance (Planck's law) to forecast emitted radiance, and it is the competence of transmitting thermal energy across the surface into the atmosphere [29].

Hence, the emissivity is calculated based on the following equation:

(3) $$\begin{array}{c} \varepsilon =\varepsilon_{\text{v}}P_{\text{v}}+\varepsilon_{\text{s}}\left(1-P_{\text{v}}\right)+d\varepsilon \;, \end{array}$$

where *ε*_v_ is the vegetative emissivity, *ε*_s_ is the soil emissivity, and *P*_v_ is the vegetation proportion [30]. According to Sobrino et al. [31], final emissivity for the Landsat 8 image is given by following equation:

(4) $$\begin{array}{c} \varepsilon =0.004P_{\text{v}}+0.989, \end{array}$$

where 0.004 is the standard deviation of 49 soil spectra, and 0.989 is the average of soil emissivity (0.97) and vegetation emissivity (0.99).

The proportion of vegetation (*P*_v_) is calculated based on the following equation [32]:

(5) $$\begin{array}{c} P_{\text{v}}={\left(\frac{\text{NDVI}-{\text{NDVI}}_{\min}}{{\text{NDVI}}_{\max}-{\text{NDVI}}_{\min}}\right)}^{2}. \end{array}$$

The following equation is used to calculate NDVI with the help of Landsat visible (band 4) and NIR (band 5) images. The amount of vegetation presence plays a major role in identifying the LST [14]:

(6) $$\begin{array}{c} \text{NDVI}=\;\frac{\text{NIR}-\text{Red}}{\text{NIR}+\text{Red}}. \end{array}$$

The final step of estimating the LST is as follows [33]:

(7) $$\begin{array}{c} \text{LST}=\frac{\text{TB}}{\left\{1+\;\left[\lambda \text{TB}/\rho \right]\text{In}\varepsilon_{\lambda }\right\}}\mathrm{{}^{\circ}C}, \end{array}$$

where *λ* is the wavelength of emitted radiance by Landsat 8 which is 10.8 (given by NASA), *ε*_*λ*_ is the land surface emissivity, and *ρ* is given by the following equation:

(8) $$\begin{array}{c} \rho =h\frac{c}{\sigma }=14388\;µ\text{m K}, \end{array}$$

where *h* is Planck's constant (6.626 × 10^−34^ Js), *σ* is the Boltzmann constant (1.38 × 10^−23^ J/K), and *c* is the velocity of light (2.988 × 10^8^ m/s) [14]. Land surface temperature for the study region has been done for November 2018 and March 2019 for comparing the LST.

NDBI is one of the significant indices used widely to identify built-up information and to extract the built-up land use with the help of band 5 which is near infrared (NIR) and band 6 which is shortwave infrared (SWIR) from the Landsat 8 satellite images using the following equation:

(9) $$\begin{array}{c} \text{NDBI}=\frac{\text{SWIR}-\text{NIR}}{\text{SWIR}+\text{NIR}}. \end{array}$$

Also, the normalized difference built-up index value lies between −1 and +1. The negative value of NDBI represents water bodies, whereas higher values represent build-up areas. NDBI value for vegetation is low. DEM (Digital Elevation model) from the ASTER remote sensing data [34] has been utilized to identify the slope of the study region with the help of ArcGIS 10.2 software. The downloaded image has gone through preprocessing of merging and extract by mask tools to delineate the study region. The land surface temperature of the study region for the two days, one in November 2018 and another in March 2019 and NDVI are also identified for the same date. NDBI is identified for the data acquired in March 2019. Finally, the slope map of the study region has been created. Randomly, 500 points have been extracted from the image through ArcGIS software, finding the corresponding values of LST_march_, NDVI_march_, NDBI_march,_ and slope with the purpose of estimating the relationship between them. The values are statistically analyzed for the creation of a model using multiple linear regression with the help of SPSS (Statistical Package for the Social Sciences).

(10) $$\begin{array}{c} Y=\alpha +\beta_{1}x_{1}+\beta_{2}x_{2}+\beta_{3}x_{3}, \end{array}$$

where *Y* is the dependent variable, *α* is the intercept, *β*_1,2,3…_ are regression coefficients of the independent variables, *x*_1,2,3_, and … are independent variables which would be the predictor of the dependent variable.

## 2. Result and Discussion

### 2.1. Land Surface Temperature

**Figure 2:**
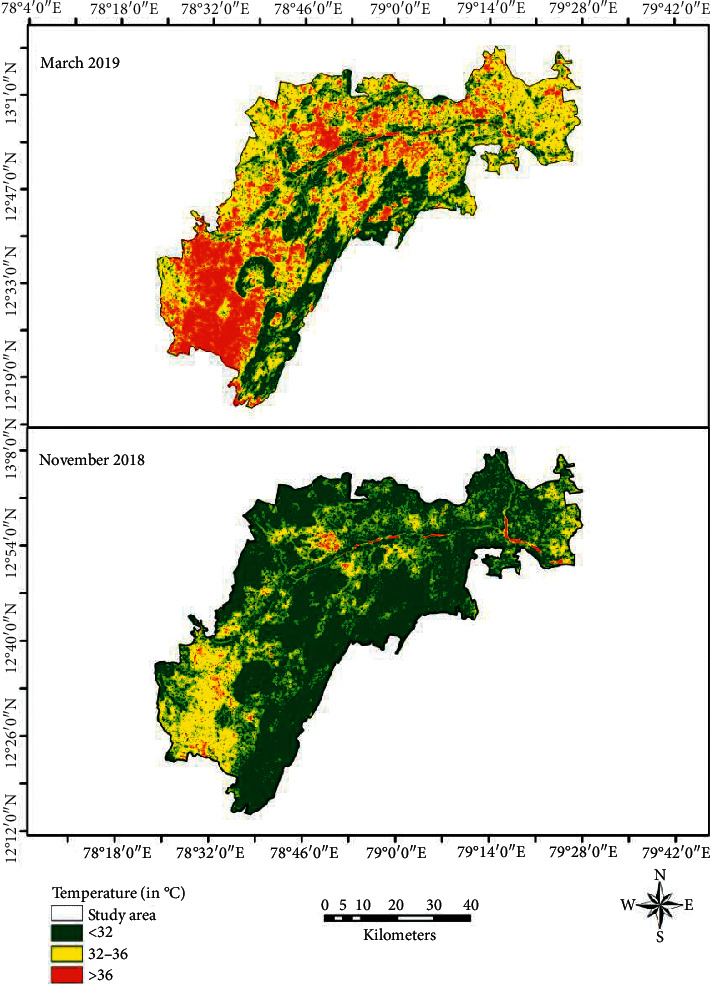
Land surface temperature.

**Figure 3:**
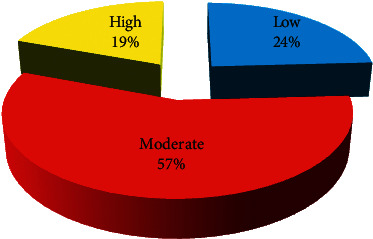
Area of LST during March.

**Figure 4:**
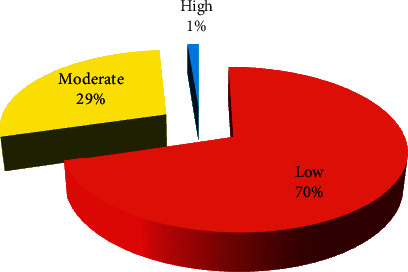
Area of LST during November.

Land surface temperature for March (Figure 2) shows that the mean temperature is 33.70°C, with a maximum temperature of 42.44°C and a minimum temperature of 24.57°C. The temperature region has been classified into 3 categories: low temperature with region lying below the 32°C, moderate temperature region lying from 32 to 36°C, and high temperature region lying where the temperature is above 36°C. 24 percent of the area comes under low-temperature region and 57 percent of the study region comes under moderate temperature. More than 19 percent of the region experiences a high temperature of above 36°C as shown in Figure 3. Maximum LST is recorded in the southwest and north-central parts of the region whereas southeast, southern, and central parts of the region have low LST. During November, the mean land surface temperature has been reduced to 30.40°C with maximum and minimum temperatures of 40.44°C and 20.78°C, respectively (Figure 2). More than 70 percent of the study region comes under low temperature (<32°C), 29 percent of the study region experiences moderate temperature, whereas only 1 percent of the region has high temperature during November (Figure 4). Most parts of the regions are low LST, expect south western parts where the LST is moderate to high.

### 2.2. NDVI, NDBI, and Slope

**Figure 5:**
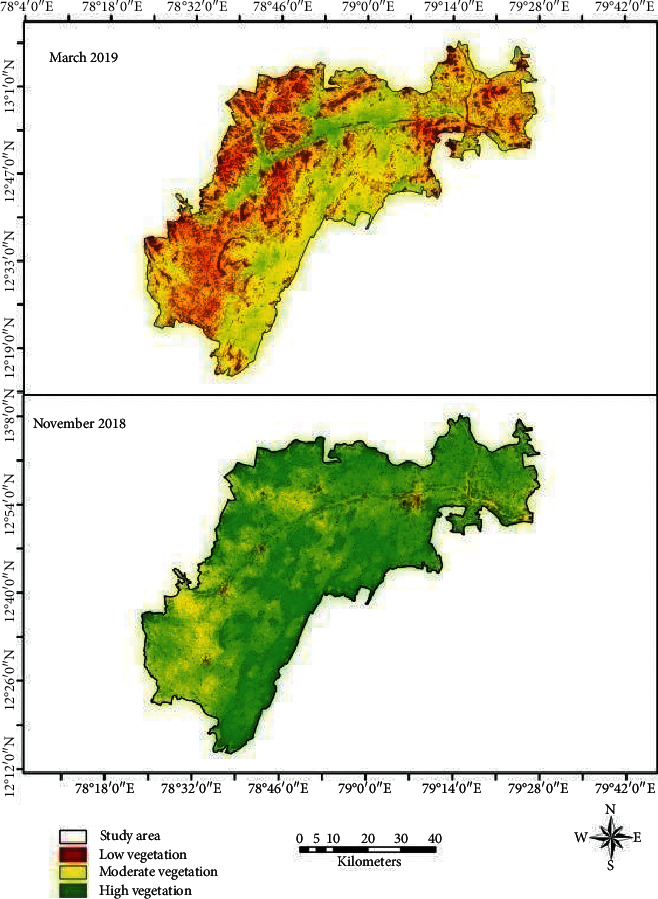
NDVI.

NDVI for March shows that the vegetation had been reduced, and only few areas in the central parts have high vegetation whereas the south and southeastern parts of the region are moderate vegetation, and northern and southwestern parts of the regions are low vegetation (Figure 5). NDVI for November has high vegetation throughout the study region except small parts of central and southwestern parts of the region. NDBI indicates that maximum built-up land is in the southwestern and north-central parts of the study region. Southern and southwestern and northeastern parts have low built-up land.

**Figure 6:**
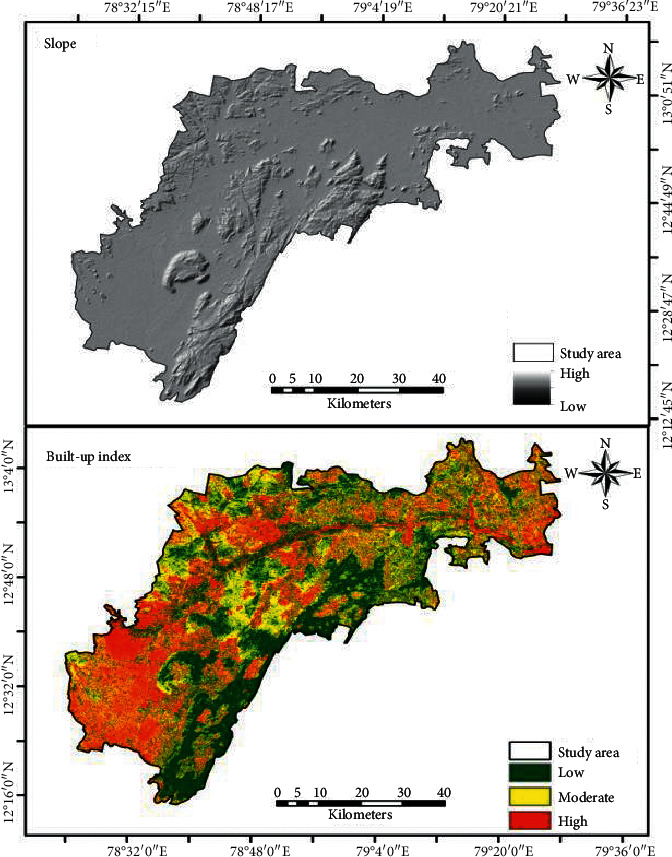
Slope and built-up index.

Regarding the gradient of the study region, the stretch of northeast to southwest parts is hilly region and small parts of the north are high elevated regions (Figure 6).

**Figure 7:**
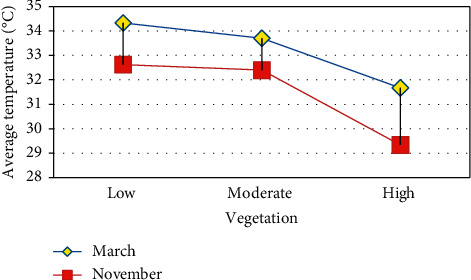
NDVI vs. LST.

**Figure 8:**
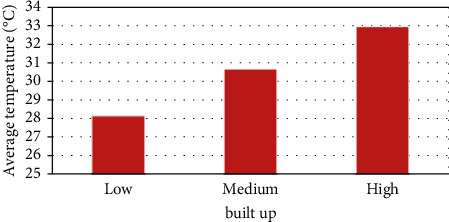
NDBI vs. LST.

**Figure 9:**
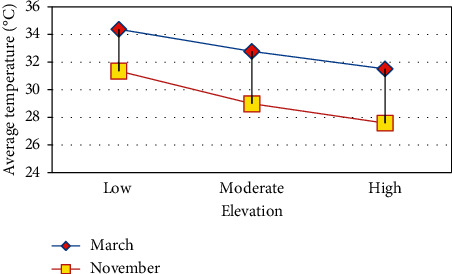
Slope vs. LST.

The analysis clearly shows that where the region experiences high vegetation, the land surface temperature is less and vice versa. Similarly, high built-up land experiences high LST and low built-up land experiences low LST. The high elevated region has low LST and vice versa. The temperature has decreased from low elevated region to high elevated region. Figures 7–9 shows that vegetation increases the LST and these increases are indirectly proportional [35, 36] built-up increases. LST also increases which is directly proportional. Height increases the temperature decreases [37–39]. Built-up land plays a major role in raising the temperature [40] because of the hard concrete surface which contains almost nil water storage which leads to less humidity. The low humidity results in slow transpiration of the land surface. This process initiates the land surface temperature to increase easily.

### 2.3. Multiple Regressions

Multiple regression model has been utilized to predict the variable for measuring land surface temperature. Here, the land surface temperature is taken as a dependent variable. NDVI, NDBI, and slope are taken as independent variables for predicting the land surface temperature of any given region. *R* is multiple correlation coefficients which are considered as a measure of the worth of the prediction of the dependent variables. The *R* value of 0.680 indicates a good level of prediction. The coefficient of determination is represented by *R* square which shows the proportion of variance in the dependent variables that can be explained by the independent variables. The *R* square value is 0.463; therefore, above 46.3% of the variation in the land surface temperature (dependent variable) is explained by NDBI, NDVI, and slope (independent variables) shown in Table 3.  Table 4 shows the analysis of variance, which shows the overall regression model is a good fit for the given data.

The significant value of 0.000 is lesser than the alpha value of 0.05, which indicates that the independent variables are statistically significant for the prediction of the dependent variable, *F* (3, 496) = 142.271, *p* < 0.05 which means the adopted regression model is a good fit of the data.

Table 5 shows the unstandardized coefficient (*B*), which tells the relationship between the land surface temperature and other independent variables. The negative value of NDVI and slope indicates that the land surface temperature increase, which decreases in vegetation and slope, so LST is negatively related to NDVI and slope.

The positive *B* value of NDBI indicates that an increase in built-up land will increase the temperature which indicates that LST is positively related to NDBI. NDVI (*t* = −3.76, *p* < 0.05), slope (*t* = −7.312, *p* < 0.05), and NDBI (*t* = 12.516, *p* < 0.05) are significant predictors of land surface temperature. From the magnitude of the *t*-statistics, we conclude that built-up land had more impact on the LST confirmed by standardized coefficients. The model also tells that with one unit increase in the vegetation, the temperature would decrease with 6 units; similarly, with one unit increase in the slope, there would be a decrease of 0.077 units in the LST. Similarly, one unit increase in the built-up land would be an increase of 14 units in the LST.

Therefore, the general form of the equation to predict land surface temperature from NDVI, slope, and NDBI is as follows:

(11) $$\begin{array}{c} \text{LST}=37.412-\left(6.363\times \text{NDVI}\right)-\left(0.077\times \text{Slope}\right)+\left(14.749\times \text{NDBI}\right). \end{array}$$

## 3. Conclusion

The study concludes that Landsat 8 images are highly useful for assessing the LST, NDVI, and NDBI. The high LST is recorded in the southwestern and central parts where there are low vegetation, high built-up land, and low elevation. LST has indirect proportion to vegetation [41, 42] and slope [37, 43, 44] but direct proportion to built-up land [45–47]. The multiple regression model is very useful for the responsible predictor of land surface temperature. The present study has adopted only three parameters (slope, NDVI, and NDBI). These all represent only 46 percent to decide the land surface temperature. The model concludes that the built-up land becomes a serious threat to the increase in land surface temperature. The study also concludes that further parameters like soil moisture, humidity, etc. should be included in order to improve the model. Vegetation plays a most significant role in mitigating the increasing land surface temperature, and built-up land would be one of the chief responsible sources for the increase of temperature. So we do not have the option to reduce the built-up land as the population is growing. The only way to mitigate this risk is to increase vegetation in the built-up land which can considerably reduce the land surface temperature [38, 47].

## Figures and Tables

**Table 1:** Details of satellite image data [25, 26].

Data	Resolution	Date
Landsat 8 OLI_TIRS	30 m	22 March 2019
Landsat 8 OLI_TIRS	30 m	14 November 2018
ASTER GDEM	1 arc-second	11 October 2011

Source: USGS, 2011, 2018, 2019

**Table 2:** Metadata for the Landsat 8 [25].

Constant	Factor	Band	Value
*M*_*L*_	Rescaling factor	10	0.000342
*A*_*L*_	Rescaling factor	10	0.1
K_1_	Thermal constant	10	1321.08
K_2_	Thermal constant	10	777.89
*O*_*i*_	Correction	10	0.29

Source: USGS, 2018, 2019.

**Table 3:** Model summary.

Model	*R*	*R* square	Adjusted *R* square	Std. error of the estimate
1	0.680^a^	0.463	0.459	1.7470475

a: predictors—(constant), NDBI, NDVI, slope. b: dependent variable—land surface temperature.

**Table 4:** Anova.

Model	Sum of squares	df	Mean square	*F*	Sig.
Regression	1302.706	3	434.235	142.271	0.000^b^
Residual	1513.879	496	3.052		
Total	2816.585	499			

a: dependent variable—land surface temperature. b: predictors—(constant), NDBI, NDVI, and slope.

**Table 5:** Coefficients.

Model	Unstandardized coefficients		Standardized coefficients	*t*	Sig.	Collinearity statistics	
	*B*	Std. error	Beta			Tolerance	VIF
(Constant)	37.412	0.321		116.474	0.000		
NDVI	−6.363	1.691	−0.140	−3.763	0.000	0.784	1.275
Slope	−0.077	0.010	−0.275	−7.312	0.000	0.765	1.307
NDBI	14.749	1.178	0.490	12.516	0.000	0.708	1.413

a: dependent variable—land surface temperature.

## Data Availability

Landsat 8 OLI_TIRS for November 2018 and March 2019 was acquired from https://earthexplorer.usgs.gov/ASTER GDEM and for October 2011 from https://earthdata.nasa.gov/.
